# Different distribution of malaria parasite in left and right extremities of vertebrate hosts translates into differences in parasite transmission

**DOI:** 10.1038/s41598-020-67180-6

**Published:** 2020-06-23

**Authors:** Romain Pigeault, Julie Isaïa, Rakiswendé S. Yerbanga, Kounbobr R. Dabiré, Jean-Bosco Ouédraogo, Anna Cohuet, Thierry Lefèvre, Philippe Christe

**Affiliations:** 1 Department of Ecology and Evolution, CH-1015 Lausanne, Switzerland; 2 Institut de Recherche en Sciences de la Santé, Bobo-Dioulasso, Burkina Faso; 3 Unité MIVEGEC, IRD 224-CNRS 5290-Université Montpellier, Montpellier, France

**Keywords:** Malaria, Parasitic infection, Ecology

## Abstract

Malaria, a vector-borne disease caused by *Plasmodium spp*., remains a major global cause of mortality. Optimization of disease control strategies requires a thorough understanding of the processes underlying parasite transmission. While the number of transmissible stages (gametocytes) of *Plasmodium* in blood is frequently used as an indicator of host-to-mosquito transmission potential, this relationship is not always clear. Significant effort has been made in developing molecular tools that improve gametocyte density estimation and therefore prediction of mosquito infection rates. However a significant level of uncertainty around estimates remains. The weakness in the relationship between gametocyte burden, measured from a blood sample, and the mosquito infection rate could be explained by a non-homogeneous distribution of gametocytes in the bloodstream. The estimated gametocyte density would then only be a single snapshot that does not reflect the host infectivity. This aspect of *Plasmodium* infection, however, remains largely neglected. In both humans and birds, we found here that the gametocyte densities differed depending on which side of the body the sample was taken, suggesting that gametocytes are not homogeneously distributed within the vertebrate host. We observed a fluctuating asymmetry, in other words, the extremity of the body with the highest density of parasites is not always the same from one individual to another. An estimation of gametocyte density from only one blood sample, as is commonly measured, could, therefore, over- or underestimated the infectivity of gametocyte carriers. This might have important consequences on the epidemiology of the disease since we show that this variation influences host-to-mosquito transmission. Vectors fed on the least infected body part had a lower parasite burden than those fed on the most infected part. The heterogeneous distribution of gametocytes in bloodstream should be considered to improve diagnosis and test new malaria control strategies.

## Introduction

The World Health Organisation estimates that in 2018, there were 228 million cases of malaria worldwide, resulting in more than 405 000 deaths^1^. African countries are disproportionally affected, and while the region experienced a 50% decline in malaria-related mortality between 2000 and 2015, the 2017 and 2018 estimates indicate a recent increase in the number of malaria cases in several countries.

In Africa and elsewhere, control strategies have focused on reducing malaria transmission through early diagnosis and treatment as well as vector control^2^. The efficacy of these interventions is however continually challenged and threatened by the evolution of insecticide^3,4^ and drug resistances^5,6^. To overcome resistance issues, the re-emergence of the concept of malaria transmission-blocking strategies^7–10^ has boosted the research efforts to find vaccines^11,12^, molecules^13,14^ or microorganisms^15–17^ able to inhibit the transmission of parasites or to disturb the life cycle of *Plasmodium* in the mosquito vector. This vector-borne parasite is also found infecting many other terrestrial vertebrate species, including other mammals, reptiles and birds and may have negative impact on vertebrate host populations^18–20^. A thorough understanding of the fundamental processes underlying the transmission of the parasite from the vertebrate host to the mosquito vector is essential to develop transmission-blocking strategies, but also to understand the dynamic of infection in natural populations.

Mosquito blood meal volumes range from 1.5 to 4µl^21,22^ and must contain at least one gametocyte (sexual stage) of each sex to result in successful transmission to the mosquito vector. The likelihood of mosquito infection appears to be mainly dictated by gametocyte density^23,24^, estimates of which are often used as an indicator of vertebrate host-to-mosquito transmission potential^25–27^. Robust estimation of gametocyte density and its relationship with the likelihood of mosquito infection are, therefore, essential to the identification of the host infection reservoir. While in human and rodent malaria systems the development of sensitive molecular techniques has significantly improved the detection, quantification, and possible sex determination of gametocytes^23,25,28,29^, the temporal and spatial dynamics of gametocyte distributions in the vertebrate host, and effects on successful transmission remain relatively neglected. These two important aspects of the dynamics of *Plasmodium* infection may partly explain why the relationship between gametocyte densities measured from blood samples and the infection rates of mosquitoes is often tenuous (Fig. S1, Table S1).

Gametocyte densities are usually estimated from a single blood sample taken from a single location in the body (e.g., finger prick or antecubital venous blood for human, wing vein and tail for bird and rodent respectively, references in Table S1) at a single point in time. Such spatiotemporal snapshots inevitably fail to capture the complex temporal and spatial dynamics of infection within the vertebrate host^30,31^. For example, a recent study found that rodent malaria *P. chabaudi* gametocytes are twice as infective at night despite being less numerous in the blood^32^. Similarly, a periodic, late afternoon increase in parasitemia, coinciding with a peak in biting activity by the mosquito vector, is observed in the avian malaria system^33^. It has also been shown that when infected birds were exposed to mosquito bites during a short period of time (3 hours), parasite transmission from host-to mosquitoes increased gradually with the biting order of vectors^34^.

Regarding spatial distribution in the vertebrate host, while mature gametocytes have long been considered to be passively displaced by the blood flow^35–37^, suggesting a random or even homogeneous distribution in the peripheral circulatory compartment^38–41^, a report of their overdispersion in mosquitoes which fed on three naturally-infected volunteers suggests an aggregated, and not homogeneous, distribution^42,43^. Furthermore, by reviewing the literature, we show that the proportion of studies showing a positive relationship between gametocyte density and transmission of the parasite to the mosquito increases significantly when mosquitoes are fed by an artificial membrane compared to mosquitoes fed directly on the skin of an infected host (Fig. S1, Table S1), a result possibly explained by a lack of heterogeneity in gametocyte spatial distribution in artificial feeding systems. Lastly, gametocyte densities may vary among different body compartments. A handful of studies have reported a higher density of gametocytes in capillary than in venous blood^44–48^ but see^49–51^. A single study estimating gametocyte densities from the same blood compartment (veins or capillaries) but from different parts of the body, found a 3-fold higher prevalence of gametocytes in arm capillary blood compared to capillary blood collected from a finger-prick^52^.

To date, no study has empirically examined the distribution of *Plasmodium* gametocytes in the peripheral blood compartment of the vertebrate host and the impact on parasite transmission. Our specific aims in this study were to answer two questions. First, are *Plasmodium* gametocyte densities similar (homogenous) between two blood samples taken at the same time but from different body parts of the vertebrate host? If not, we hypothesize that a heterogeneous distribution of gametocyte densities could be detected through random deviations from a symmetric distribution between different body parts (i.e. fluctuating asymmetry^53^). Second, does vertebrate host-to-mosquito transmission of *Plasmodium* vary according to the location of mosquito bites? If gametocyte density is heterogeneous in the bloodstream, we predicted a higher transmission rate in higher infected body part. This work uses both human (*P. falciparum/Anopheles gambiae s.s*) and avian malaria (*P. relictum/Culex pipiens*) systems to measure gametocyte density at two different body locations: the left and right hand in humans and the left and right leg in birds. The presence of directional and/or fluctuating asymmetry was then investigated while taking into account the measurement variations. Due to ethical reasons, the effect of mosquito bite location on parasite transmission was carried out only with the avian malaria system. Avian malaria is the oldest experimental model for investigating the life cycle of *Plasmodium* parasites and is an ideal surrogate for understanding the biology of human malaria parasites^54,55^.

## Results

### Spatial heterogeneity of Plasmodium infection in the vertebrate host

#### Human malaria

**Figure 1:**
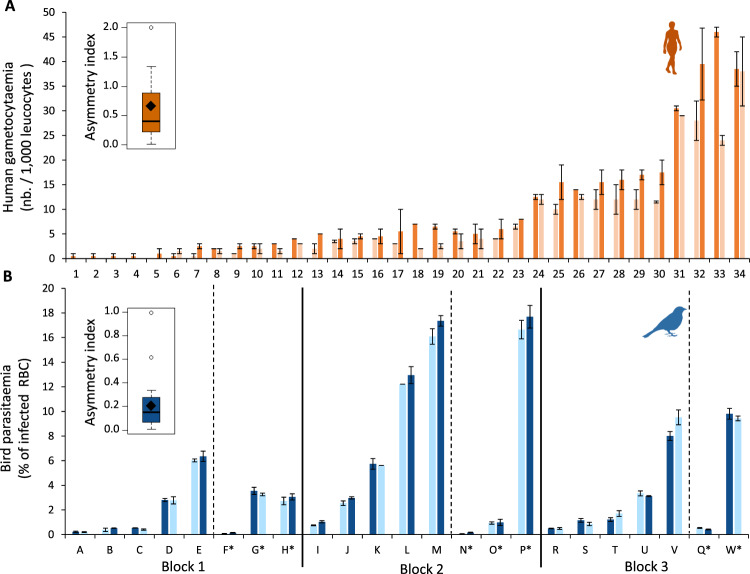
Variation in infection density between two body parts. (**A**) Variation in human gametocyte density (nb. of gametocytes gametocytes per 1000 leucocytes) between the left hand (left bar) and the right hand (right bar). (**B**) Variation in bird parasitaemia (% of infected red blood cells) between the left leg (left bar) and the right leg (right bar). The black vertical lines on panel B separates the three experimental blocks (see materials & methods). Each number (humans) or letter (birds) corresponds to one individual. The * for individuals to the left of the vertical dotted line in each block corresponds birds unexposed to mosquito bites. Light colored bars correspond to the body part with the lower gametocyte density (human) or parasite density (bird), and dark colored bars correspond to the body part with higher densities. Error bars represent standard error around the mean. Boxplots represent the individual asymmetry index corrected for size-dependence. Boxes above and below the medians (horizontal lines) show the first and third quartiles, respectively. Black diamond represent the means.

Gametocyte densities, from the right and left hands of each volunteer, were determined by two independent microscopists. The repeatability of measurements between observers was highly significant (R = 0.914, CI = 0.866, 0.945, p < 0.0001) and the variation in gametocyte densities within each extremity of each individual represented only 0.01% of the variance. On the other hand, heterogeneity between infected individuals explained a large part of the variance (89%, Fig. 1A). Regarding variation in gametocyte densities between human extremities, while the analysis showed absence of directional asymmetry (i.e. it was not always the same hand, right or left, that had the highest gametocyte density, F = 0.52, P = 0.474), a fluctuating asymmetry was observed (χ^2^_1_ = 4.50, P = 0.033, Fig. 1A, Fig. S2A). Fluctuating asymmetry explained 2.4% of the total variance in gametocyte density. Although the asymmetry analysis considers the measurement variations, this result may be largely influenced by the individuals showing extremely low intensities of infection. However, when individuals with one or less than one gametocyte on average per 1000 leucocytes per hand, and then individuals with 5 or less than 5 gametocytes, were removed from the dataset, fluctuating symmetry was still detected (χ^2^_1_ = 5.88, P = 0.015, χ^2^_1_ = 4.23, P = 0.039, respectively). The individual asymmetry index corrected for size-dependence averaged 0.67 ± 0.11 for the complete dataset, 0.36 ± 0.05 for the dataset containing only individuals with more than one detected gametocytes and 0.25 ± 0.05 for the dataset containing only individuals with more than 5 detected gametocytes.

#### Avian malaria

For each leg of each bird, three independent measurements of parasitaemia were made from three different drops of blood. The measurements repeatability between the three blood smears was highly significant (R = 0.987 CI = 0.980, 0.992, p < 0.0001). Variation in parasite densities within each leg of each bird represents only 0.03% of the variance. As for human malaria, heterogeneity between birds explains a large part of the variance (97%, Fig. 1B). While we showed absence of directional asymmetry (F = 2.45, P = 0.135), a fluctuating asymmetry of parasitaemia between the two bird sides was observed (1.7% of the variance, χ^2^_1_ = 10.81, P = 0.001, Fig. 1B, Fig. S2B). When individuals with a parasitaemia of less than 0.1% where removed from the dataset, the fluctuating asymmetry of parasite density was still detected (χ^2^_1_ = 9.052, P = 0.002). However, when individuals with a parasitaemia of less than 0.5% where removed from the dataset, the fluctuating asymmetry was no longer detected (χ^2^_1_ = 1.014, P = 0.314). The individual asymmetry index corrected for size-dependence averaged 0.21 ± 0.04 for the full dataset, 0.17 ± 0.03 for the dataset containing only birds with a parasitaemia of more than 0.1% and 0.13 ± 0.02 for the dataset containing only birds with a parasitaemia of more than 0.5%.

### Parasite transmission to mosquito vectors

To investigate the effect of the location of mosquito bites on *Plasmodium* transmission, the left and right legs of infected birds (*Serinus canaria*) were independently and simultaneously exposed to mosquitoes (*Culex pipiens*) for 3 hours. Immediately following exposure to mosquitoes, blood from both legs was collected to measure parasite densities (see above), and each leg was classified as either lower infected leg (LIL) or higher infected leg (HIL). Blood-fed mosquitoes were dissected one week post blood meal to count the number of parasites in their midgut (oocyst stage).

**Figure 2:**
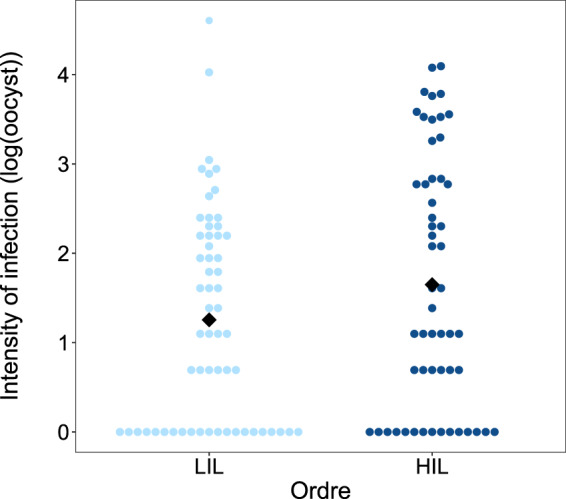
Oocyst burden in mosquitoes fed on either the lower infected leg (LIL) or on the higher infected leg (HIL). Black diamond represent the means.

We found no significant differences among mosquitoes fed on the lower (LIL) or on the higher infected leg (HIL) in either the proportion of females taking a blood meal (mean ± s.e., HIL = 0.44 ± 0.05, LIL = 0.36 ± 0.04; χ^2^_1_ = 3.03 P = 0.082), blood meal size (HIL = 15.01 ± 0.67, LIL = 14.57 ± 0.63; χ^2^_1_ = 0.795 P = 0.373), or infection prevalence (proportion of mosquitoes containing at least 1 oocyst, HIL = 0.40 ± 0.04, LIL = 0.41 ± 0.04; χ^2^_1_ = 0.005 P = 0.994). The analysis of oocyst burden included only mosquitoes having at least one oocyst in the midgut. We found that infected females who fed on the HIL had a significantly higher oocyst burden than females fed on the LIL (HIL = 12.70 ± 2.18 vs. LIL = 7.79 ± 1.93; χ^2^_1_ = 5.24 P = 0.022, Fig. 2).

## Discussion

Reducing *Plasmodium* transmission from the vertebrate host to the insect vector is a critical component of global efforts to control malaria^56^. Understanding the processes underlying the relationship between *Plasmodium* gametocyte densities and mosquito infection is, therefore, crucial to assess the effectiveness of control programs and their effects on transmission. An essential first step is to obtain accurate estimations of gametocyte densities in an infected host. To this end, new molecular tools have been developed to detect and estimate more precisely gametocyte densities, including densities of males and females. It is noteworthy that these methods have significantly improved the prediction of mosquito infection rates^23,51^. Nevertheless, a significant level of uncertainty in predicting mosquito infection rates remains.

Another factor potentially affecting the development of better predictors of parasite transmission to the mosquito host is variation in the spatial distribution of mature gametocytes within the vertebrate host^42,43^. Although largely ignored, a non-homogeneous distribution of the parasite in the blood could bias the estimation of the vertebrate host infectivity. In the vast majority of studies, gametocyte densities are exclusively estimated from a blood sample taken from a single body location^24,55,57–61^. The objective of our study was to assess variation in parasite densities among different locations of a similar organ (hand or leg) at a given timestamp (i.e., sampling time), rather than to specifically measure differences between the right and left extremities of individuals. Here, we showed that the gametocyte densities differed depending on which side of the body the sample was taken, suggesting that gametocytes are not homogeneously distributed within the vertebrate host. In both humans and birds, we observed a fluctuating asymmetry, in other words it was not always the same body extremities, right or left, that had the highest parasite density.

The differences in parasite load observed between the extremities of vertebrate hosts may impact the transmission of *Plasmodium* to mosquitoes. Indeed, in the birds we observed that mosquitoes fed on the least infected body part had a lower parasite burden than those fed on the most infected part. In turn, variation in oocyst density could have epidemiological consequences as recent work suggested that increased parasite burden in mosquitoes can result in increased probability of transmission to vertebrate hosts^62,63^. Consequently, using a single measure of gametocyte density from a single blood sample taken from only one side of the body, may not provide an accurate estimate of a host’s infectivity. Our results suggest that the most efficient way to obtain a more accurate estimate of gametocyte density, and, therefore, a better predictor of infectiousness, would be to combine several independent density measurements from different body parts.

The mechanisms leading to the establishment of a non-homogeneous distribution of *Plasmodium* in the blood of the vertebrate host are unknown. Gametocytes are not motile and cannot actively migrate to accumulate in the capillaries. Passive accumulation of gametocytes in some sub-dermal capillaries could induce a non-homogeneous distribution of *Plasmodium* in the vertebrate host. For instance, the elongated asymmetric curvature of *P. falciparum* gametocytes may facilitate their blockage in the dermal capillaries^64^. Mature gametocyte aggregation might also partly explain the spatial heterogeneity in the distribution of gametocytes^42,65^. Active aggregation mechanisms potentially involving binding interactions between infected red blood cells containing late developmental stages of gametocytes have however not been observed in either human or avian malaria parasites (“rosetting-like” adhesion^51,66^). Nevertheless, a study described a very unusual clustering behavior between *Leucocytozoon toddi* gametocytes (another haemosporidian parasite). Authors observed significant proportions of male and female gametocytes in aggregations involving substantial contact^67^.

When the gametocyte density is low, an adaptive strategy allowing the aggregation of several sexual stages of *Plasmodium* within a blood capillary or even within a same red blood cell^68^ may increase the probability that a mosquito will be infected^42,43,69^. In this case, while the majority of blood-fed mosquitoes did not ingest any parasite, those biting an area containing aggregated gametocytes will be undoubtedly infected by malaria. However, the benefits of such a strategy are reduced once gametocyte density increases to a level ensuring the successful transmission regardless of the mosquito biting site. In this case, homogeneous distribution should maximize transmission^43^. Thus, a plasticity in the level of aggregation in response to changes in gametocyte density would allow for optimization of transmission to the mosquito vector throughout the infection.

Although our data did not allow us to test this prediction we observed that the asymmetry index does indeed seem to be lower in hosts with higher parasite densities. In addition, in the avian malaria system, when individuals with low gametocytaemia (<0.5%) were removed from the analysis, the fluctuating asymmetry was no longer detected. The distribution of parasites within highly infected birds therefore appeared to be more homogeneous than that observed in the lowest infected hosts. Nevertheless, another explanation could be that the non-homogeneous distribution of *Plasmodium* gametocytes detected in weakly infected birds is an artifact which could be due to a stochastic effect (*i.e*. the chance to detect very few parasites regardless of the body location). Although in human subjects, fluctuating asymmetry was always detected even after removing individuals with low gametocyte loads, it should be noted that only one blood sample was taken at each hand (due to ethical reasons). Gametocyte density was estimated by two independent microscopists and our analyses consider measurement variations but it cannot be ruled out that by increasing the number of blood samples taken from each extremity of humans, the difference between the two locations will diminish. Nevertheless, whether the difference in parasite density observed here is due to heterogeneity in the distribution of gametocytes between extremities or to stochastic effects during sampling, in both cases it would affect the mosquitoes when they take blood meals and thus increase the heterogeneity of vector exposure.

Given that malaria infection is temporally dynamic^30,33^, the single measurement used in this study to compare the number of parasites between different sides of the body does not allow us to determine whether the apparent non-homogeneous distribution changes over time and is simply a single snapshot of an underlying process that is more complex and dynamic. A logical next step is to monitor gametocyte densities at different body parts with repeated measurements over the course of the infection. Furthermore, of particular relevance would be to compare gametocyte densities among different body locations in regard to variation in mosquito attraction to these sites. For instance, it is known that the major vectors of *P. falciparum* (*An. gambiae s.s*., *An. arabiensis*, *An. funestus*) all have a strong preference for feeding close to the ground which is associated to increased biting rate on legs, ankles, and feet^70,71^. However, a recent study of 8 infected donors found no significant increase in gametocyte density estimated from leg skin biopsies compared to that from arm skin as would be predicted^51^.

Improving the detection and estimation of gametocyte density in infected hosts is fundamental to improve the diagnosis of gametocyte carriers and therefore identify infectious reservoirs but also to develop and test new malaria control strategies. In this study, we found that the gametocyte burden varies between different body parts. We argue that it is essential to collect several blood samples from different body parts to depict accurately gametocyte density and infectiousness. We further propose that such sampling, combined with a better understanding of mosquito biting preferences, may better our understanding of within-host malaria infection dynamics and, ultimately, the fundamental processes underlying parasite transmission from human-to-mosquito.

## Materials and Methods

### Human malaria

The study was conducted at the Institut de Recherche en Sciences de la Santé in Bobo Dioulasso, South-Western Burkina Faso. The intensity of malaria transmission is high and perennial in this area, with a peak from August to November. Blood slides were collected from December 2018 to July 2019 from 42 asymptomatic children aged 5–12 years attending the elementary schools of Dandé, Soumousso, Klesso, Samandeni - four villages located in the surroundings of Bobo Dioulasso. *P. falciparum* is the predominant parasite species in these villages, accounting for more than 95% of malaria cases^72^.

Separate finger-prick blood samples from the right and left hand of each volunteer were collected, Giemsa-stained and screened for asexual parasites and gametocytes. Gametocyte densities were determined from each slide as the number of gametocytes per 1000 leucocytes. Each slide was read twice by two independent qualified microscopists^72^. Slides were declared negative after a minimum reading of 100 fields. We discovered 8 gametocyte-free individuals leaving 34 individuals for analysis.

### Avian malaria

#### Parasite strain

*Plasmodium relictum* is the most prevalent form of avian malaria in Europe^73^. The lineage used in these experiments (lineage SGS1) was isolated from infected great tits (*Parus major*) caught in the region of Lausanne (Switzerland) in 2015. The strain has since been maintained by regular passage between infected and naïve canaries (*Serinus canaria*) via intraperitoneal injection. Twenty three uninfected canaries were split into three experimental blocks (Block 1: 8, Block 2: 8, Block 3: 7) and inoculated by means of an intraperitoneal injection of 150–200 μL of a blood mixture collected from five chronically infected canaries. Birds in the same experimental block were infected with a blood mixture from an independent group of infected birds. For each block infected birds were then either “exposed” (block 1, 2, 3 = 5) or “unexposed” (block 1, 2 = 3, block 3 = 2) to mosquito bites (Fig. 1B).

#### Mosquito rearing

*Culex pipiens* mosquitos used in the experiment were from a population collected from the field (Lausanne, 46°31′25.607″N 6°34′40.714″E, altitude: 380 m) in August 2017 and since maintained under laboratory conditions. Mosquitoes were reared as described by Vézilier *et al*.^74^ in an insectary at 25 °C ± 1 °C, 70 ± 5% RH and with 12 L:12D photoperiod. On the day prior to mosquito exposure, 500 7–10 day old female mosquitoes were haphazardly chosen from different emergence cages and placed inside new cages (100 females per cage). During this time females were deprived of sugar solution to increase hunger levels and maximize the biting rate. Water was provided to prevent dehydration, but removed 6 hours prior to the start of the experiment.

#### Experimental design

The three experimental blocks were carried out in February, March and April 2018 respectively. Twelve days after infection, coinciding with the acute phase of the *P. relictum* infection in canaries^55^, birds were placed individually into compartmentalized cages designed for physically separating their two legs. For birds in the “exposed” group, at 6:00 pm, 45–50 uninfected female mosquitoes were added to each compartment (left and right) for 180 minutes. Unexposed birds were placed under the same experimental conditions but without mosquitoes. At the end of the mosquito exposure period (9:00 pm), a red lamp was used to capture mosquitoes and five microliters of blood was collected from the medial metatarsal vein of each leg. Three independent drops of blood were then smeared onto three different microscope slides for each of the samples. Blood fed mosquitoes were placed individually into a numbered plastic tube covered with a mesh. Food was provided in the form of a cotton pad soaked in a 10% sugar solution placed on top of each tube. Mosquitoes were dissected 7 to 8 days later and the number of *Plasmodium* oocysts in their midgut counted with the aid of a binocular microscope^74^. Haematin excreted at the bottom of each plastic tube was quantified as an estimate of the female’s blood meal size^74^.

The intensity of bird infection (parasitaemia) was determined visually by counting the number of infected red blood cells per 3000 erythrocytes in randomly chosen fields on the blood smears^73^. The three replicate were used to calculate an average parasitaemia (mean ± SE) for each leg of each bird. The legs of each bird were then classified as either the lower infected leg (LIL) or higher infected leg (HIL). All slides were examined by the same experimenter, and parasitaemia was used as a proxy for transmissible stage (gametocytes) production because parasitaemia and gametocytaemia are strongly positively correlated in this system (see Fig. 2 in^55^).

### Statistical analyses

Analyses were carried out using the R statistical package (v. 3.4.1, http://www.cran.r-project.org/).

### Repeatability estimation

The repeatability estimation of the parasite load between observers, in the case of samples from humans, and between slides within the same extremities, in the case of birds, were obtained using the intra-class correlation coefficient (package rptR^75^).

### Asymmetry of parasite densities

The presence of directional and/or fluctuating asymmetry (DA and FA, respectively), while taking sampling or measurement variation into account, for avian and human malaria system respectively, was tested by the restricted maximum-likelihood (REML) estimation of a mixed regression model where we fitted the models to the repeated measurements of right and left parasite density sides^76^. Side (right or left) was included as fixed effects in the models and log-transformed trait values (gametocyte number or parasitaemia) as response variables, individual identity and side were fitted as a random effects. This procedure allows the separation of measurement error from bilateral asymmetry analysis^76^. The presence of DA was assessed by the F-test of the fixed effect (side^76^), with degrees of freedom corrected for statistical dependence by Satterthwaite formulae^77^. We used random intercepts and the fixed slopes (estimated within individuals) to estimate the variation in individual trait value and the individual FA, respectively. The significance of FA was then calculated by performing a likelihood ratio test (LRT) comparing two models: the original full model and a reduced model without the side as a random effect^76^.

The presence of antisymmetry was excluded after inspection of the distributions of the right (R) minus left (L) side values for gametocyte densities and parasitaemias in humans and birds, respectively (Kolmogorov-Smirnov test, Human: D = 0,229, P = 0,057; Bird D = 0,199, P = 0,260^53,78^). As our results indicated the existence of fluctuating asymmetry in human and avian malaria system (see Results), we calculated an individual asymmetry index (AI) as the unsigned right-left difference (|R-L | ) between the average of trait values across the two (human) or three (bird) replicate counts of each individual^53,79^. Trait size dependence was examined with a non-parametric test of association (Spearman test) between unsigned AI values (|R − L | ) and (R + L)/2. These analyses revealed positive trait size dependence for both human and avian malaria system (human: S = 2956, p = 0.0009, Rho = 0.55; bird: 384, p < 0.0001, Rho = 0.83). We therefore calculated a new asymmetry index with a correction for size-dependence (AIcor = |R − L | /(R + L)/2 [63,65]).

The part of variance explained by difference in counts between observers or repeated slides, for humans and birds respectively, was estimated from a mixed model with observers or repeated slides as a fixed factor, and side (left, right), nested in individual, as a random factor (MuMin package^80^). The part of variance explained by both heterogeneities between host and by FA were estimated from the maximal model described previously to test the presence of DA or FA.

### Parasite transmission to mosquito vector

Mixed effects models were used to analyze the effect of bird leg infection class (LIL or HIL) on the mosquito blood meal rate (proportion of females that had taken a blood meal), blood meal size and *Plasmodium* transmission to the vector. The explanatory variables leg class (LIL or HIL) and haematin (when it was appropriate) were fitted as fixed factors. Individual, nested within experimental block, was fitted as a random factor. Blood meal rate and infection prevalence (oocyst presence/absence) were analyzed using GLMM with a binomial error distribution (lme4 package^81^). Blood meal size and mosquito infection intensity (number of oocysts) were analyzed using lmer with normal error distribution. For the analysis of infection intensity, only individuals that developed ≥ 1 oocyst were included. Maximal models, including all higher-order interactions, were simplified by eliminating non-significant terms and interactions to establish a minimal model^82^. Non-significant interactions and terms were removed step by step according to their significance using either a likelihood ratio test or an F test^83^. The significant Chi-square or F values given in the text are for the minimal model, whereas non-significant values correspond to those obtained before the deletion of the variable from the model.

### Ethics statement

Human blood samples were collected prior to treatment with a dose of artemether–lumefantrine according to National Malaria Control Programme recommendation and after written informed consent was obtained from the parent(s) or guardian(s). Ethical clearance was provided by the national ethics committee of Burkina Faso (no. 2018-9-118) and the institutional committee of the Institut de Recherche en Sciences de la Santé (no. A06-2018/CEIRES). The methods used in this study were performed in accordance with the relevant guidelines of the national ethics committee of Burkina Faso and the institutional committee of the Institut de Recherche en Sciences de la Santé. The animal care and protocols used in the avian malaria study was approved by the Ethical Committee of the Vaud Canton veterinary authority, Switzerland (authorization number 1730.4). The methods used in this study were performed in accordance with the relevant guidelines and regulations of the Vaud Canton veterinary authority (Switzerland).

## Supplementary information

Supplementary information.

## Data Availability

All data supporting the conclusions of this paper are available on the Dryad website: 10.5061/dryad.6djh9w0z5.
