# Supplementary material for: Different distribution of malaria parasite in left and right extremities of vertebrate hosts translates into differences in parasite transmission
Source: Sci Rep. 2020 Jun 23;10:10183. doi: 10.1038/s41598-020-67180-6 (PMC7311528; doi:10.1038/s41598-020-67180-6)
Supplement: Supplementary file 1 — Supplementary information. [file 41598_2020_67180_MOESM1_ESM.docx]

**Different distribution of malaria parasite in left and right extremities of vertebrate hosts translates into differences in parasite transmission**

Romain Pigeault ^1*^, Julie Isaïa ^1^, Rakiswendé S. Yerbanga ^2^, Kounbobr R. Dabiré ^2^, Jean-Bosco Ouédraogo ^2^, Anna Cohuet ^3^, Thierry Lefèvre ^2,3^, Philippe Christe ^1^

^1^ Department of Ecology and Evolution, CH-1015 Lausanne, Switzerland

^2^ Institut de Recherche en Sciences de la Santé, Bobo-Dioulasso, Burkina Faso

^3^ Unité MIVEGEC, IRD 224-CNRS 5290-Université Montpellier, Montpellier, France

*Corresponding author: romain.pigeault@unil.ch

**Supporting information**

- **Fig S1**
- **Fig S2**
- **Table S1**

**
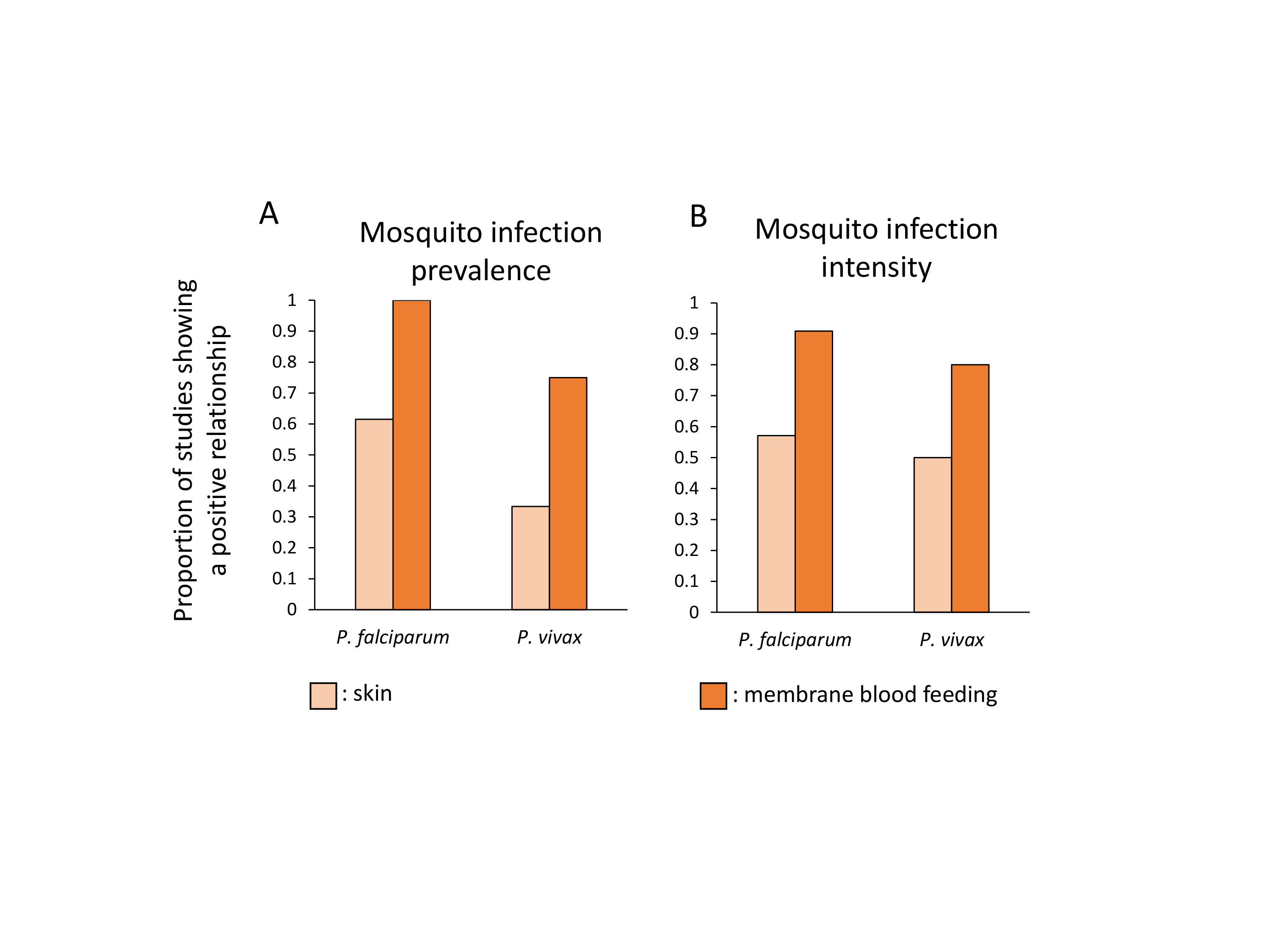
**

**Fig S1.** Proportion of studies showing a positive relationship between gametocyte density, estimated from human blood, and (A) mosquito infection prevalence or (B) oocyst burden. The light-orange bars represents mosquitoes fed directly on the skin of infected individuals, the dark-orange bars represents mosquitoes fed with artificial membrane feeding. See Table S1 for references.


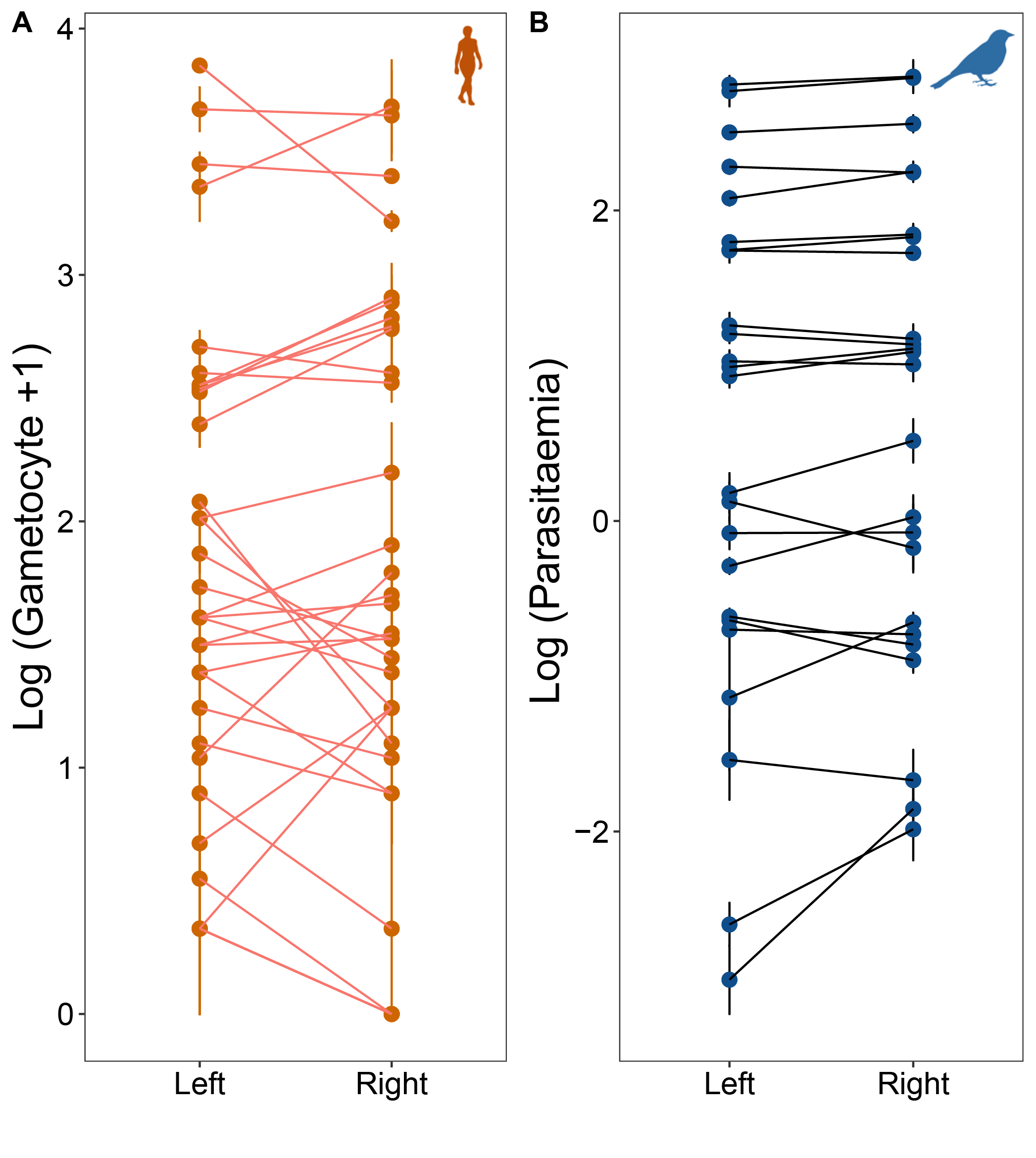


**Fig S2.** Fluctuating asymmetry of parasite density in vertebrate host. (A) Gametocyte densities in the left and right hands of infected humans. (B) Parasitaemia in the left and right legs of infected birds. Each dot represents an individual (mean ± s.e.).

**Table S1:** Studies investigating the relationship between gametocyte densities, estimated from human blood, and mosquito infection prevalence and/or oocyst burden.

| **Host** | **Feeding_Method** | **Vector_species** | **Parasite_species** | **Prevalence_in_mosquitoes** | **Infection_intensity_in_mosquitoes** | **Refs** |
| --- | --- | --- | --- | --- | --- | --- |
| Human | direct | *A. quadrimaculatus* | *P. falciparum* | Lake of correlation | Lake of correlation | [1] |
| Human | direct | *A. gambiae* | *P. falciparum* | Positive correlation | NA | [2] |
| Human | direct | *A. quadrimaculatus* | *P. falciparum* | Positive correlation | NA | [3] |
| Human | direct | *A. stephensis* | *P. falciparum* | Positive correlation | NA | [4] |
| Human | direct | *A. balabacensis* | *P. falciparum* | Positive correlation | NA | [4] |
| Human | indirect | *A. gambiae* | *P. falciparum* | Positive correlation | NA | [5] |
| Human | indirect | *A. farauti* | *P. falciparum* | Positive correlation | Positive correlation | [6] |
| Human | direct | *A. tessellatus* | *P. falciparum* | Positive correlation | Positive correlation | [7] |
| Human | indirect | *A. gambiae* | *P. falciparum* | Positive correlation | Lake of correlation | [8] |
| Human | indirect | *A. gambiae* | *P. falciparum* | Positive correlation | Positive correlation | [9] |
| Human | indirect | *A. gambiae* | *P. falciparum* | Positive correlation | NA | [10] |
| Human | indirect | *A. gambiae* | *P. falciparum* | Positive correlation | Positive correlation | [11] |
| Human | direct | *A. gambiae* | *P. falciparum* | Lake of correlation | Lake of correlation | [12] |
| Human | direct | *A. funestus* | *P. falciparum* | Lake of correlation | Lake of correlation | [12] |
| Human | indirect | *A. gambiae* | *P. falciparum* | Positive correlation | Positive correlation | [13] |
| Human | direct | *A. gambiae* | *P. falciparum* | Positive correlation | Positive correlation | [14] |
| Human | indirect | *A. gambiae* | *P. falciparum* | Positive correlation | Positive correlation | [14] |
| Human | indirect | *An. arabiensis* | *P. falciparum* | Positive correlation | NA | [15] |
| Human | direct | *A. gambiae* | *P. falciparum* | Lake of correlation | NA | [16] |
| Human | direct | *A. minimus* | *P. falciparum* | Lake of correlation | Lake of correlation | [17] |
| Human | indirect | *A. gambiae* | *P. falciparum* | Positive correlation | Positive correlation | [18] |
| Human | indirect | *A. gambiae* | *P. falciparum* | Positive correlation | NA | [19] |
| Human | direct | *A. gambiae or A. arabiensis* | *P. falciparum* | Positive correlation | Positive correlation | [20] |
| Human | indirect | *A. gambiae or A. arabiensis* | *P. falciparum* | Positive correlation | Positive correlation | [20] |
| Human | direct | *An. Arabiensis* | *P. falciparum* | Positive correlation | NA | [21] |
| Human | indirect | *An. coluzzii* | *P. falciparum* | Positive correlation | Positive correlation | [22] |
| Human | indirect | *An. coluzzii* | *P. falciparum* | Positive correlation | Positive correlation | [23] |
| Human | indirect | *An. coluzzii* | *P. falciparum* | Positive correlation | Positive correlation | [24] |
| Human | direct | *A. stephensis* | *P. vivax* | Lake of correlation | NA | [4] |
| Human | direct | *A. balabacensis* | *P. vivax* | Lake of correlation | NA | [4] |
| Human | direct | *A. tessellatus* | *P. vivax* | Positive correlation | Positive correlation | [7] |
| Human | direct | *A. dirus* | *P. vivax* | Positive correlation | Positive correlation | [25] |
| Human | direct | *A. dirus* | *P. vivax* | Lake of correlation | Lake of correlation | [26] |
| Human | direct | *A. minimus* | *P. vivax* | Lake of correlation | Lake of correlation | [17] |
| Human | indirect | *An. darlingi* | *P. vivax* | Positive correlation | Positive correlation | [27] |
| Human | indirect | *An. sinensis* | *P. vivax* | NA | Positive correlation | [28] |
| Human | indirect | *An. anthropophagus* | *P. vivax* | NA | Positive correlation | [28] |
| Human | indirect | *An. darlingi* | *P. vivax* | Positive correlation | Positive correlation | [29] |
| Human | indirect | *An. aquasalis* | *P. vivax* | Positive correlation | Positive correlation | [29] |
| Human | indirect | *An. triannulatus* | *P. vivax* | Lake of correlation | Lake of correlation | [29] |
| Human | indirect | *An. albitarsis s.l.* | *P. vivax* | Lake of correlation | Lake of correlation | [29] |
| Human | indirect | *An. pharoensis* | *P. vivax* | Positive correlation | Positive correlation | [30] |
| Human | indirect | *An. arabiensis* | *P. vivax* | Positive correlation | Positive correlation | [30] |
| Human | indirect | *An. aquasalis* | *P. vivax* | Positive correlation | Positive correlation | [31] |
| Monkey | direct | *A. macultatus* | *P. inui* | Positive correlation | Lake of correlation | [32] |
| Monkey | direct | *A. macultatus* | *P. inui* | Lake of correlation | Positive correlation | [32] |
| Monkey | direct | *An. stephensi* | *P. cynomolgi* | Lake of correlation | Lake of correlation | [33] |
| Rodent | direct | *A.farauti* | *P.berghei* | Lake of correlation | Lake of correlation | [34] |
| Rodent | direct | *A. stephensis* | *P.berghei* | NA | Lake of correlation | [35] |
| Rodent | indirect | *An. stephensi* | *P. berghei* | Positive correlation | Positive correlation | [24] |
| Rodent | direct | *An. stephensi* | *P. chabaudi* | NA | Positive correlation | [36] |
| Rodent | direct | *A. stephensis* | *P. chabaudi* | Positive correlation | NA | [37] |
| Rodent | direct | *An. stephensi* | *P. chabaudi* | Positive correlation | NA | [38] |
| Avian | direct | *A. aegypti* | *P.gallinaceum* | NA | NA | [39] |
| Avian | direct | *A. aegypti* | *P. gallinaceum* | NA | Positive correlation | [40] |
| Avian | indirect | *A. aegypti* | *P. gallinaceum* | NA | Positive correlation | [40] |
| Avian | direct | *A. aegypti* | *P. gallinaceum* | NA | Lake of correlation | [41] |
| Avian | direct | *A. albopictuss* | *P. fallax* | NA | Negative correlation | [41] |
| Avian | direct | *A. albopictuss* | *P. fallax* | NA | Negative correlation | [41] |
| Avian | direct | *A. albopictuss* | *P. fallax* | NA | Positive correlation | [41] |
| Avian | direct | *A. albopictuss* | *P. fallax* | NA | Lake of correlation | [41] |
| Avian | direct | *C. pipiens* | *P. cathemerium* | NA | Lake of correlation | [41] |
| Avian | direct | *C. tarsali* | *P. cathemerium* | NA | Linear correlation | [41] |
| Avian | direct | *A. aegypti* | *P. gallinaceum* | NA | Negative correlation | [42] |
| Avian | direct | *A. quadrimaculatus* | *P. gallinaceum* | NA | Negative correlation | [42] |
| Avian | direct | *C. pipiens* | *P.relictum* | Lake of correlation | Positive correlation | [43] |
| Lizard | direct | *L. vexator* | *P. mexicanum* | Positive correlation | Positive correlation | [44] |

**References**

1. Young MD, Hardman NF. 1948 The infectivity of native malarias in South Carolina to *Anopheles quadrimaculatus*. *Am. J. Trop. Med. Hyg.* **28**, 303–311.

2. Draper CC. 1953 Observations on the infectiousness of gametocytes in hyperendemic malaria. *Transactions of the Royal Society of Tropical Medicine and Hygiene* **47**, 160–165. (doi:10.1016/0035-9203(53)90072-8)

3. Jeffery GM, Eyles DE. 1955 Infectivity to mosquitoes of *Plasmodium* *falciparum* as related to gametocyte density and duration of infection. *Am. J. Trop. Med. Hyg.* **4**, 781–789.

4. Rutledge LC, Gould DJ, Tantichareon B. 1969 Factors affecting the infection of anophelines with human malaria in Thailand. *Transactions of the Royal Society of Tropical Medicine and Hygiene* **63**, 613–619. (doi:10.1016/0035-9203(69)90180-1)

5. Graves PM. 1980 Studies on the use of a membrane feeding technique for infecting *Anopheles gambiae* with *Plasmodium falciparum*. *Trans. R. Soc. Trop. Med. Hyg.* **74**, 738–742.

6. Graves PM *et al.* 1988 Measurement of malarial infectivity of human populations to mosquitoes in the Madang area, Papua, New Guinea. *Parasitology* **96 ( Pt 2)**, 251–263.

7. Gamage-Mendis AC, Rajakaruna J, Carter R, Mendis KN. 1991 Infectious reservoir of *Plasmodium vivax* and *Plasmodium falciparum* malaria in an endemic region of Sri Lanka. *Am. J. Trop. Med. Hyg.* **45**, 479–487.

8. Boudin C, Olivier M, Molez JF, Chiron JP, Ambroise-Thomas P. 1993 High human malarial infectivity to laboratory-bred *Anopheles gambiae* in a village in Burkina Faso. *Am. J. Trop. Med. Hyg.* **48**, 700–706.

9. Tchuinkam T, Mulder B, Dechering K, Stoffels H, Verhave JP, Cot M, Carnevale P, Meuwissen JH, Robert V. 1993 Experimental infections of *Anopheles gambiae* with *Plasmodium falciparum* of naturally infected gametocyte carriers in Cameroon: factors influencing the infectivity to mosquitoes. *Trop. Med. Parasitol.* **44**, 271–276.

10. Mulder B, Tchuinkam T, Dechering K, Verhave JP, Carnevale P, Meuwissen JH, Robert V. 1994 Malaria transmission-blocking activity in experimental infections of *Anopheles gambiae* from naturally infected *Plasmodium falciparum* gametocyte carriers. *Trans. R. Soc. Trop. Med. Hyg.* **88**, 121–125.

11. Robert V, Read AF, Essong J, Tchuinkam T, Mulder B, Verhave J-P, Carnevale P. 1996 Effect of gametocyte sex ratio on infectivity of *Plasmodium falciparum* to *Anopheles gambiae*. *Trans R Soc Trop Med Hyg* **90**, 621–624. (doi:10.1016/S0035-9203(96)90408-3)

12. Haji H, Smith T, Charlwood JD, Meuwissen JH. 1996 Absence of relationships between selected human factors and natural infectivity of *Plasmodium falciparum* to mosquitoes in an area of high transmission. *Parasitology* **113**, 425–431. (doi:10.1017/S0031182000081488)

13. Drakeley CJ, Secka I, Correa S, Greenwood BM, Targett GA. 1999 Host haematological factors influencing the transmission of *Plasmodium falciparum* gametocytes to *Anopheles gambiae s.s.* mosquitoes. *Trop. Med. Int. Health* **4**, 131–138.

14. Bonnet S, Gouagna C, Safeukui I, Meunier J-Y, Boudin C. 2000 Comparison of artificial membrane feeding with direct skin feeding to estimate infectiousness of *Plasmodium falciparum* gametocyte carriers to mosquitoes. *Trans R Soc Trop Med Hyg* **94**, 103–106. (doi:10.1016/S0035-9203(00)90456-5)

15. Robert V, Awono-Ambene HP, Hesran JYL, Trape JF. 2000 Gametocytemia and infectivity to mosquitoes of patients with uncomplicated *Plasmodium falciparum* malaria attacks treated with chloroquine or sulfadoxine plus pyrimethamine. *The American Journal of Tropical Medicine and Hygiene* **62**, 210–216. (doi:10.4269/ajtmh.2000.62.210)

16. Bonnet S, Gouagna LC, Paul RE, Safeukui I, Meunier JY, Boudin C. 2003 Estimation of malaria transmission from humans to mosquitoes in two neighbouring villages in south Cameroon: evaluation and comparison of several indices. *Trans. R. Soc. Trop. Med. Hyg.* **97**, 53–59.

17. Pethleart A, Prajakwong S, Suwonkerd W, Corthong B, Webber R, Curtis C. 2004 Infectious reservoir of *Plasmodium* infection in Mae Hong Son Province, north-west Thailand. *Malar. J.* **3**, 34. (doi:10.1186/1475-2875-3-34)

18. Schneider P, Bousema JT, Gouagna LC, Otieno S, Vegte-Bolmer MVD, Omar SA, Sauerwein RW. 2007 Submicroscopic *Plasmodium Falciparum* gametocyte densities frequently result in mosquito infection. *Am J Trop Med Hyg* **76**, 470–474.

19. Ouédraogo AL *et al.* 2009 Substantial contribution of submicroscopical *Plasmodium falciparum* gametocyte carriage to the infectious reservoir in an area of seasonal transmission. *PLoS One* **4**. (doi:10.1371/journal.pone.0008410)

20. Bousema T *et al.* 2012 Mosquito feeding assays to determine the infectiousness of naturally infected *Plasmodium* *falciparum* gametocyte carriers. *PLOS ONE* **7**, e42821. (doi:10.1371/journal.pone.0042821)

21. Gaye A, Bousema T, Libasse G, Ndiath MO, Konaté L, Jawara M, Faye O, Sokhna C. 2015 Infectiousness of the human population to *Anopheles arabiensis* by direct skin feeding in an area hypoendemic for malaria in Senegal. *The American Journal of Tropical Medicine and Hygiene* **92**, 648–652. (doi:10.4269/ajtmh.14-0402)

22. Kopya E, Ndo C, Fossog BT, Tchuinkam T, Awono-Ambene P, Antonio-Nkondjio C. 2015 Pyrethroid resistance and susceptibility to *Plasmodium falciparum* infection of *Anopheles coluzzii* populations from Yaoundé (Cameroon). *Journal of Entomology and Zoology Studies* **3**, 05–10.

23. Morlais I *et al.* 2015 *Plasmodium falciparum* mating patterns and mosquito infectivity of natural isolates of gametocytes. *PLOS ONE* **10**, e0123777. (doi:10.1371/journal.pone.0123777)

24. Da DF, Churcher TS, Yerbanga RS, Yaméogo B, Sangaré I, Ouedraogo JB, Sinden RE, Blagborough AM, Cohuet A. 2015 Experimental study of the relationship between *Plasmodium* gametocyte density and infection success in mosquitoes; implications for the evaluation of malaria transmission-reducing interventions. *Experimental Parasitology* **149**, 74–83. (doi:10.1016/j.exppara.2014.12.010)

25. Sattabongkot J, Maneechai N, Rosenberg R. 1991 *Plasmodium vivax*: gametocyte infectivity of naturally infected Thai adults. *Parasitology* **102 Pt 1**, 27–31.

26. Sattabongkot J, Maneechai N, Phunkitchar V, Eikarat N, Khuntirat B, Sirichaisinthop J, Burge R, Coleman RE. 2003 Comparison of artificial membrane feeding with direct skin feeding to estimate the infectiousness of *Plasmodium vivax* gametocyte carriers to mosquitoes. *The American Journal of Tropical Medicine and Hygiene* **69**, 529–535. (doi:10.4269/ajtmh.2003.69.529)

27. Bharti AR, Chuquiyauri R, Brouwer KC, Stancil J, Lin J, Llanos-Cuentas A, Vinetz JM. 2006 Experimental infection of the neotropical malaria vecor anopheles darlingi by human patient- derived *Plasmodium vivax* in the peruvian amazon. *The American Journal of Tropical Medicine and Hygiene* **75**, 610–616. (doi:10.4269/ajtmh.2006.75.610)

28. Zhu G, Xia H, Zhou H, Li J, Lu F, Liu Y, Cao J, Gao Q, Sattabongkot J. 2013 Susceptibility of *Anopheles sinensis* to *Plasmodium vivax* in malarial outbreak areas of central China. *Parasit Vectors* **6**, 176. (doi:10.1186/1756-3305-6-176)

29. Rios-Velásquez CM *et al.* 2013 Experimental *Plasmodium* vivax infection of key Anopheles species from the Brazilian Amazon. *Malaria Journal* **12**, 460. (doi:10.1186/1475-2875-12-460)

30. Abduselam N *et al.* 2016 Similar trends of susceptibility in *Anopheles arabiensis* and *Anopheles pharoensis* to *Plasmodium vivax* infection in Ethiopia. *Parasites & Vectors* **9**, 552. (doi:10.1186/s13071-016-1839-0)

31. Martins-Campos KM *et al.* 2018 Infection of *Anopheles aquasalis* from symptomatic and asymptomatic *Plasmodium vivax* infections in Manaus, western Brazilian Amazon. *Parasit Vectors* **11**, 288. (doi:10.1186/s13071-018-2749-0)

32. Collins WE, Contacos PG, Guinn EG, Held JR. 1966 Studies on the transmission of simian malarias, I. Transmission of two strains of *Plasmodium inui* by *Anopheles maculatus* and *A. stephensi.* *J. Parasitol.* **52**, 664–668.

33. Puri SK, Dutta GP. 2005 *Plasmodium cynomolgi*: Gametocytocidal activity of the anti-malarial compound CDRI 80/53 (elubaquine) in rhesus monkeys. *Experimental Parasitology* **111**, 8–13. (doi:10.1016/j.exppara.2005.05.007)

34. Ramasamy MS, Ramasamy RJA. 1990 Effect of anti‐mosquito antibodies on the infectivity of the rodent malaria parasite *Plasmodium berghei* to *Anopheles farauti*. *Medical and Veterinary Entomology* **4**, 161–166. (doi:10.1111/j.1365-2915.1990.tb00274.x)

35. Dearsly AL, Sinden RE, Self IA. 1990 Sexual development in malarial parasites: gametocyte production, fertility and infectivity to the mosquito vector. *Parasitology* **100**, 359–368. (doi:10.1017/S0031182000078628)

36. Taylor LH, Walliker D, Read AF. 1997 Mixed-genotype infections of the rodent malaria *Plasmodium chabaudi* are more infectious to mosquitoes than single-genotype infections. *Parasitology* **115 ( Pt 2)**, 121–132.

37. Mackinnon MJ, Read AF. 1999 Genetic relationships between parasite virulence and transmission in the rodent malaria *Plasmodium chabaudi*. *Evolution* **53**, 689–703. (doi:10.2307/2640710)

38. Schneider P, Rund SSC, Smith NL, Prior KF, O’Donnell AJ, Reece SE. 2018 Adaptive periodicity in the infectivity of malaria gametocytes to mosquitoes. *Proc. Biol. Sci.* **285**. (doi:10.1098/rspb.2018.1876)

39. Cantrell W, Jordan HB. 1946 Changes in the infectiousness of gametocytes during the course of *Plasmodium gallinaceum* infections. *J. Infect. Dis.* **78**, 153–159.

40. Eyles DE. 1951 Studies on *Plasmodium gallinaceum*. Characteristics of the infection in the mosquito, *Aedes aegypti*. *Am J Epidemiol* **54**, 101–112. (doi:10.1093/oxfordjournals.aje.a119461)

41. Huff CG, Marchbank DF. 1955 Changes in infectiousness of malarial gametocytes. I. Patterns of oocyst production in seven host-parasite combinations. *Experimental Parasitology* **4**, 256–270. (doi:10.1016/0014-4894(55)90029-1)

42. Beasley SJ. 1972 Plasmodium gallinaceum: Comparative infection rates in *Aedes aegypti* and *Anopheles quadrimaculatus*. *Experimental Parasitology* **32**, 11–20. (doi:10.1016/0014-4894(72)90004-5)

43. Pigeault R, Vézilier J, Cornet S, Zélé F, Nicot A, Perret P, Gandon S, Rivero A. 2015 Avian malaria: a new lease of life for an old experimental model to study the evolutionary ecology of *Plasmodium*. *Phil. Trans. R. Soc. B* **370**, 20140300. (doi:10.1098/rstb.2014.0300)

44. Schall JJ. 2000 Transmission success of the malaria parasite *Plasmodium mexicanum* into its vector: role of gametocyte density and sex ratio. *Parasitology* **121 Pt 6**, 575–580.
